# Supplementary material for: Excitability in the p53 network mediates robust signaling with tunable activation thresholds in single cells
Source: Sci Rep. 2017 Apr 18;7:46571. doi: 10.1038/srep46571 (PMC5394551; doi:10.1038/srep46571)
Supplement: Supplementary Information [file srep46571-s1.pdf]

Excitability in the p53 network mediates robust signaling with tunable activation thresholds in single cells

Supplementary Figures

Gregor Moenke, Elena Cristiano, Ana Finzel, Dhana Friedrich,
Hanspeter Herzel, Martin Falcke and Alexander Loewer

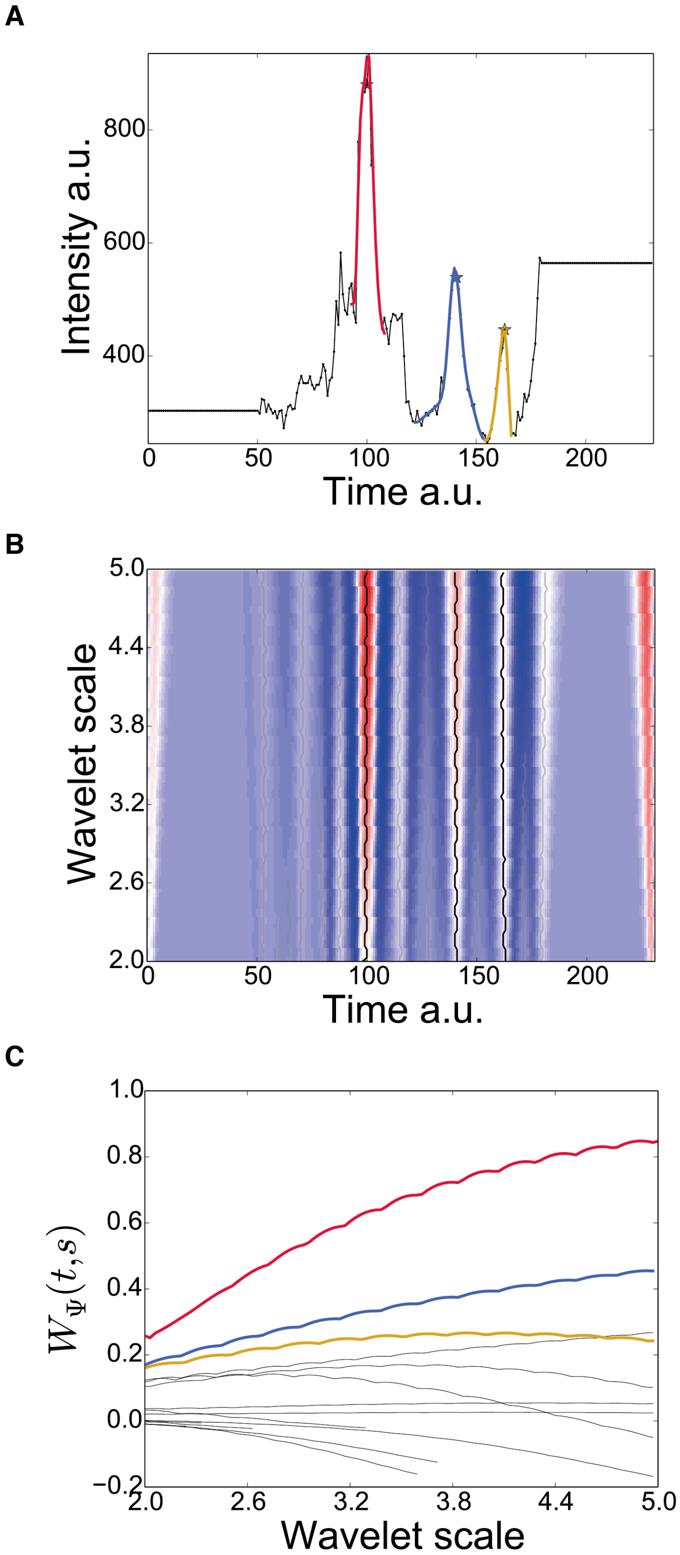

Figure S1 – Wavelet based peak detection method

A An exemplary single cell p53 trajectory, the detected pulses are color coded.

B The wavelet transform corresponding to the trajectory shown in **A.**

C The ridge lines extracted from the wavelet transform (B). The color-coding corresponds to the pulses in (A)**.** See also material and methods section in the main text.

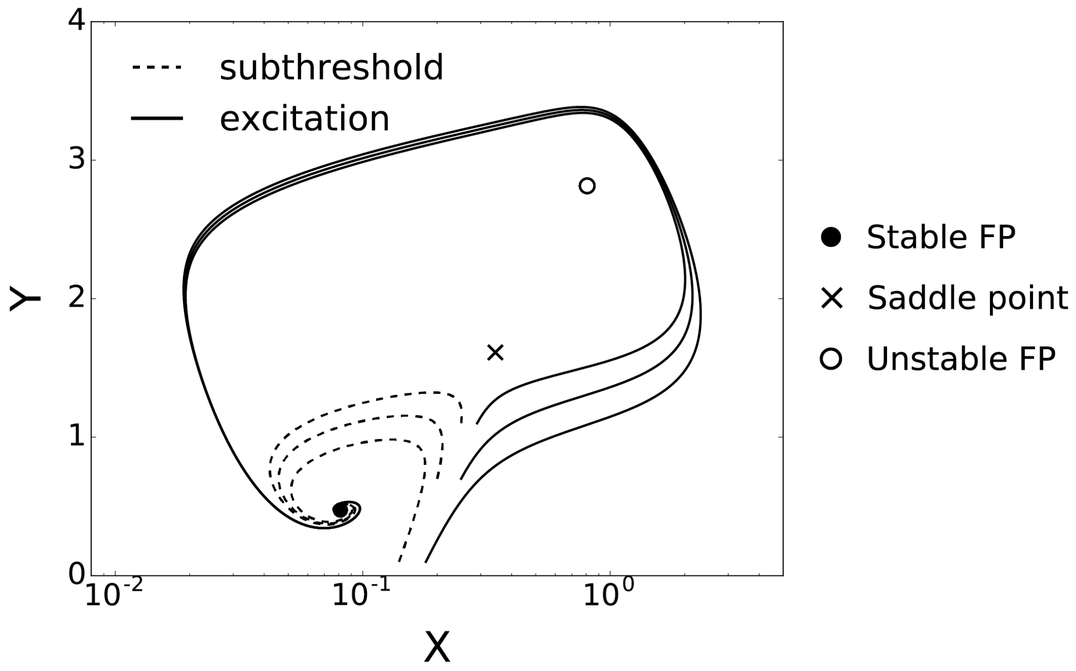

Figure S2 – Phase portrait of the excitable toy model
Three subthreshold trajectories, which rapidly settle back to the stable fixed point, and three excitation loops (pulses) making the full phase space excursion are shown for the NPF system. The direction dependent threshold visibly originates from the saddle point and separates the phase space into subthreshold and excitable dynamics. It is called a *separatrix* and coincides with the stable manifold of the saddle point. Such a phase space structure with a saddle as organizing center is typical for type I excitable systems.

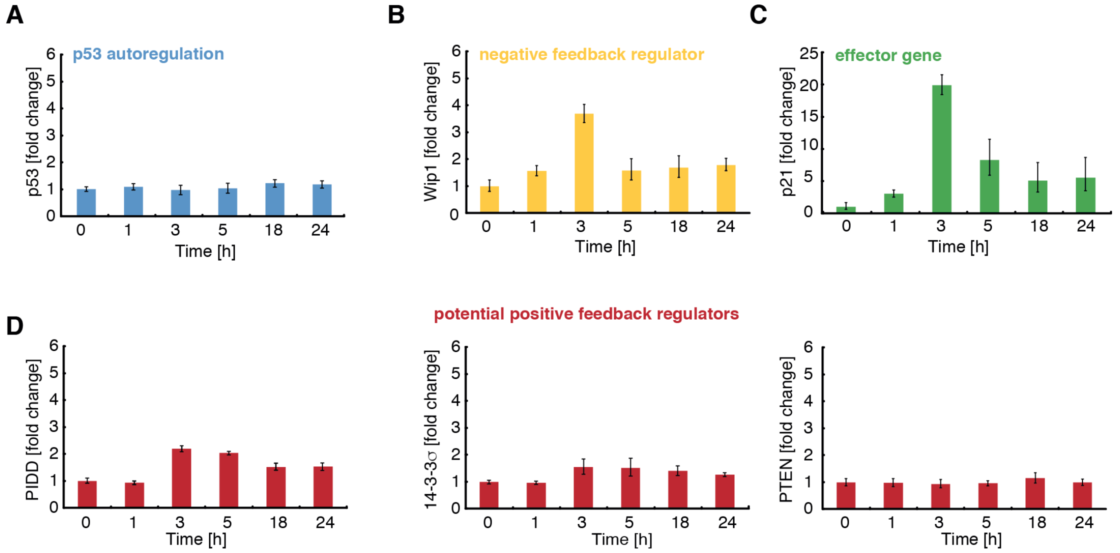

Figure S3 – qPCR quantification of potential positive feedbacks ATM inhibitor studies

Time resolved mean expression of p53 (A), the known negative feedback regulator Wip1 (B), the effector p21 (C) and potential transcriptional positive feedbacks (D) as indicated on the y-axis. Timing and fold change of the three potential feedbacks PIDD, 14-3-3 sigma and PTEN do not support an effect on p53 pulse formation. Error bars indicate standard deviation.

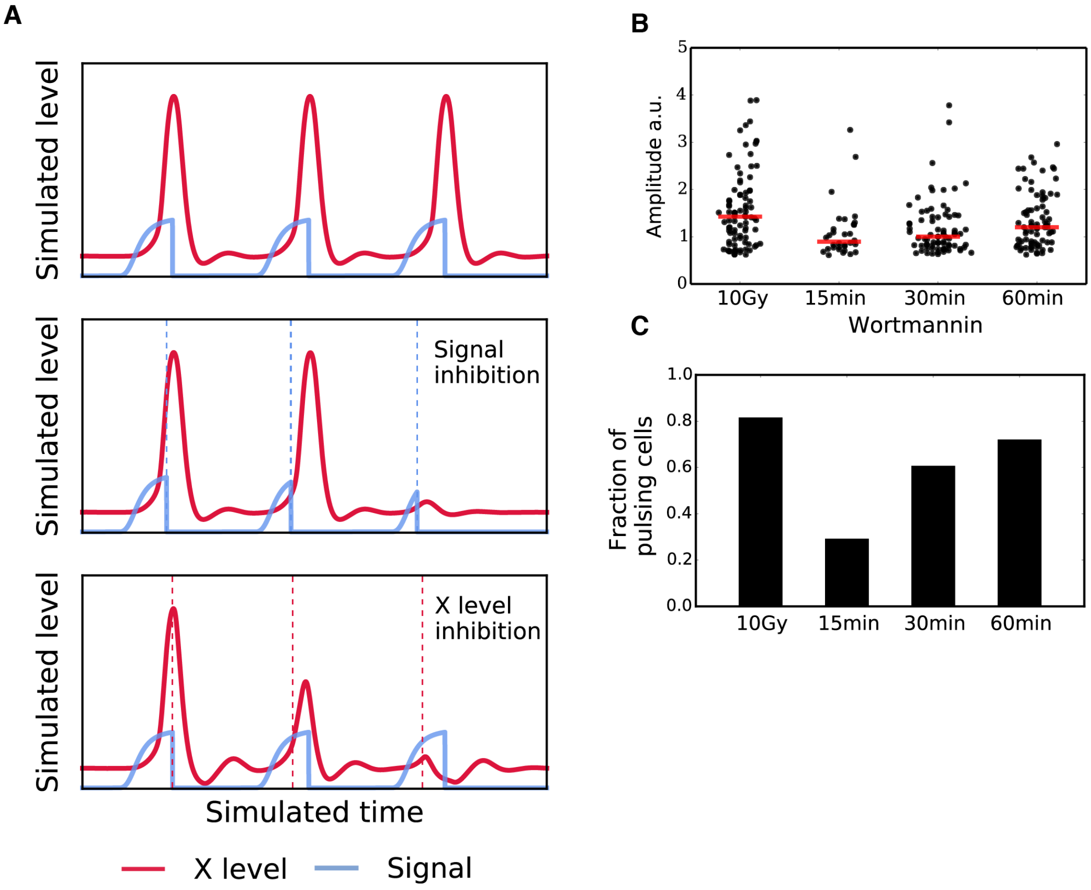

Figure S4 – ATM inhibitor studies

A Differential effect of signal vs. species X inhibition for the generic negative-positive feedback (NPF) system presented in Figure 2 of the main text. Signal inhibition leads to an "all-or-none" systems response. X inhibition leads to a time-of-inhibition dependent collapse of the excitation loop, reflected by the smaller response amplitudes.

B Single cell p53 first pulse amplitudes after strong stimulation (10Gy) and kinase inhibitor Wortmannin addition at time points as indicated. The earlier ATM is inhibited, the smaller are the detected p53 pulses.

C Fraction of responsive cells for the experimental conditions described in **F**. Early ATM inhibition abrogates the p53 pulse formation for a substantial amount of cells below the detection limit.

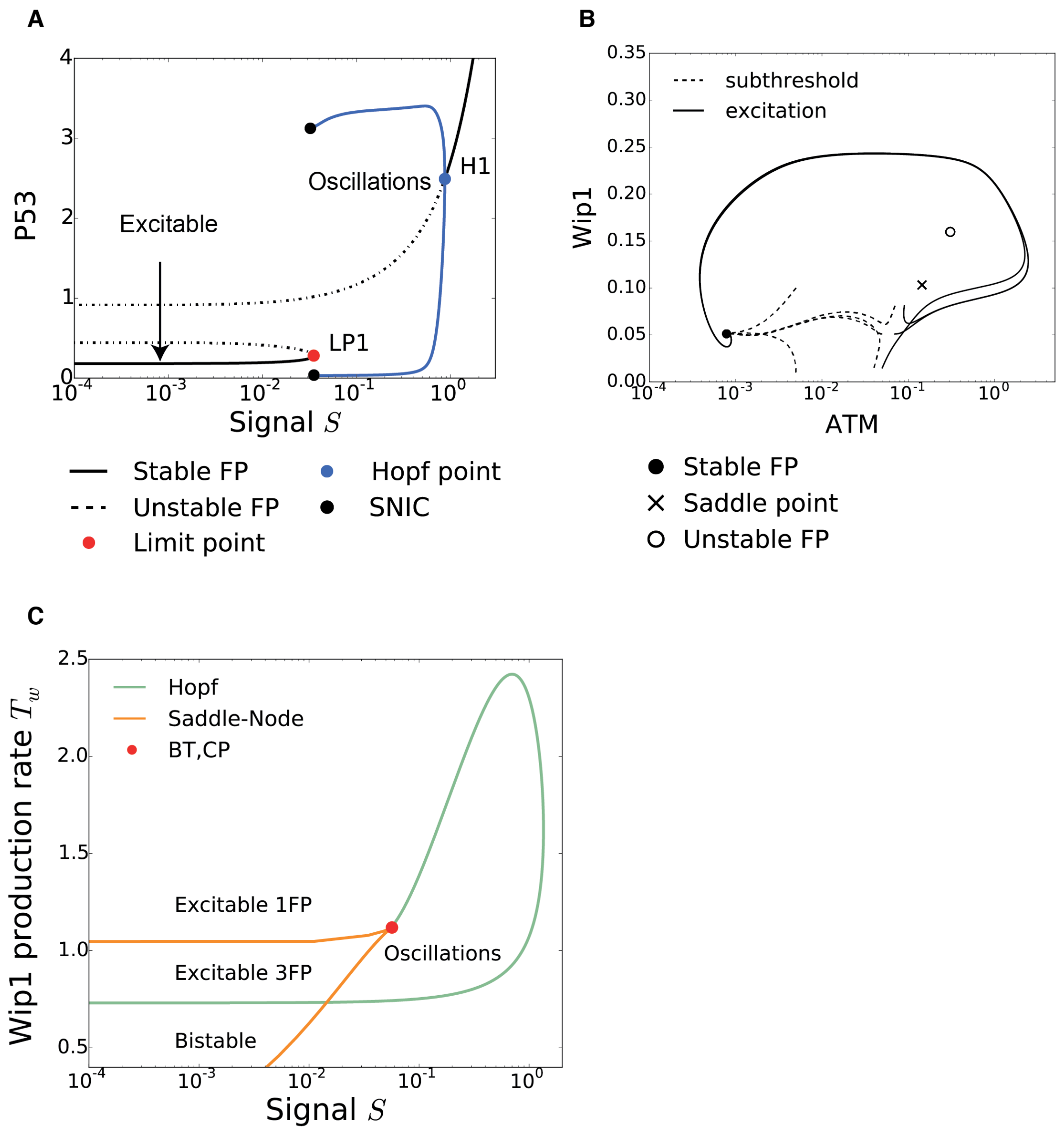

Figure S5 – Bifurcation analysis of the p53 model

A One parameter bifurcation analysis. The control parameter is the stimulus strengths (S). For low stimulation the system is in the excitable regime. Upon the simultaneous homoclinic (SNIC) and saddle-node bifurcation (LP1) a limit cycle suddenly appears with full amplitude. The arrow marks the point in parameter space for the phase portrait shown in **B** See also Supplementary Text.

B Phase portrait for the p53 model in the excitable regime as marked in **A.** Shown are five subthreshold trajectories which rapidly settle back to the stable fixed point and three excitation loops (pulses) making the full phase space excursion. Neither high or low Wip1 concentrations alone can trigger a pulse, only strong ATM activation crosses the threshold.

C Two parameter bifurcation analysis, the first control parameter is the stimulus strengths (S), the second parameter Wip1 protein maturation rate (T_W). The Cusp Point (CP) spawns two saddle-node curves which enclose the excitable regime. A Bogdanov-Takens Point (BT) is very close to the Cusp Point and spawns a Hopf curve enclosing the oscillatory regime. For the one fixed-point regime a response was classified as excitable if it shows at least a five-fold ATM response upon a small initial perturbation. See also Supplementary Text.

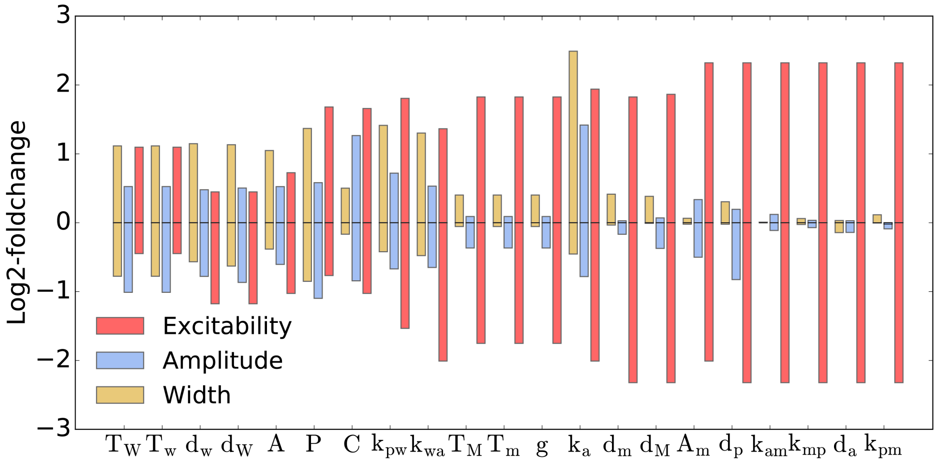

Figure S6 – Local sensitivity analysis of the deterministic core model

Model parameter variations are given as log2 fold change from the standard values given in supplementary section 4. The red bars indicate the extent of the excitable regime in parameter space. It was determined by stimulating the system with a perturbation in ATM^* and checking for a pulsatile response. Within the excitable regime, the maximal and minimal effect of the respective parameter variations on the p53 pulse amplitude (width) is indicated in blue (yellow). Parameters associated with the Wip1 phosphatase show the highest sensitivity, which underlines its proposed function as a major modulator of the p53 signaling pathway.

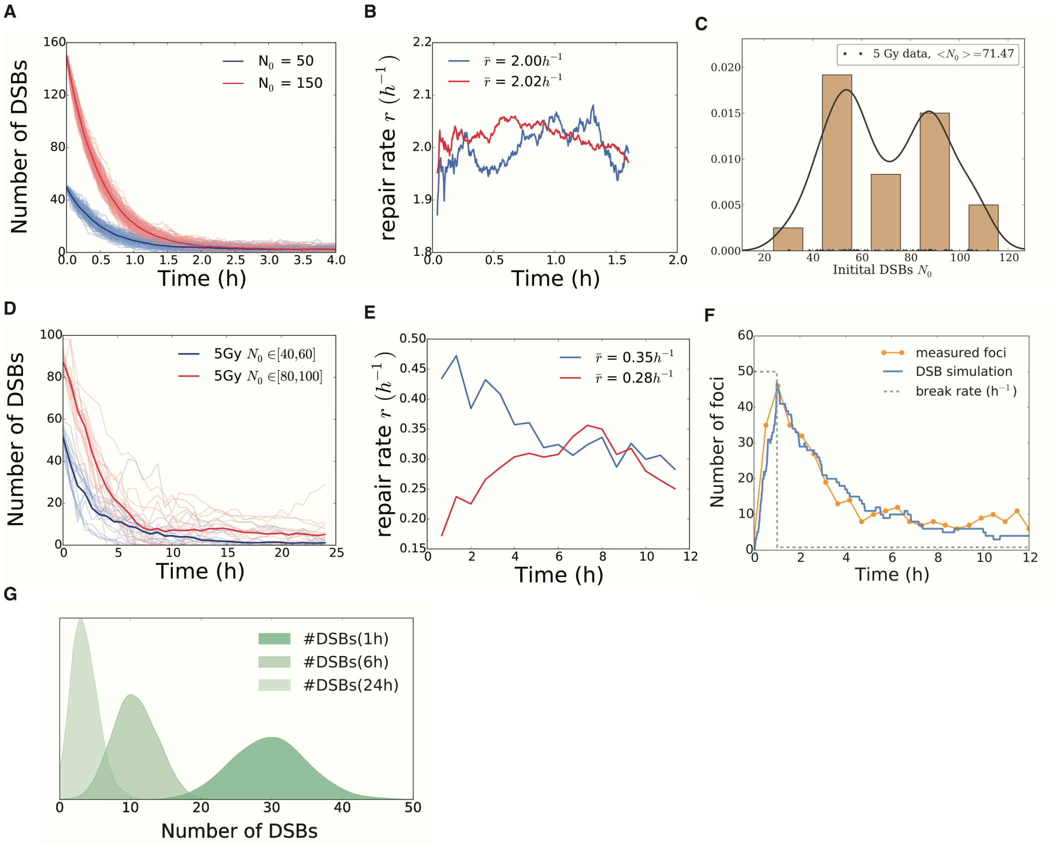

Figure S7 – Parameter estimation for the stochastic double strand break (DSB) process

A Synthetic ensembles of the time-homogeneous DSB process, with two different initial DSB numbers as indicated.

B Estimation of the repair rate for the two synthetic ensembles shown in (A) with the method described in the Supplementary Text.

C Distribution of the initial number of DSBs for the experimental data used for parameter estimation.

D Measured DSB dynamics in single cells, data is binned as indicated. N=67

E Repair rate estimation carried out as in (B) for the measured trajectories.

F Exemplary realization of the time-inhomogeneous DSB process as described in Supplementary Text. The break rate switches from the high stimulation rate to the low basal rate.

G For medium stimulation (initial break rate = 30 DSB/h) and 5000 realizations the number-densities of the DBSs are shown for three different time points. At all times, but especially at the initial DSB induction, there is a high variability in the number of DSBs.

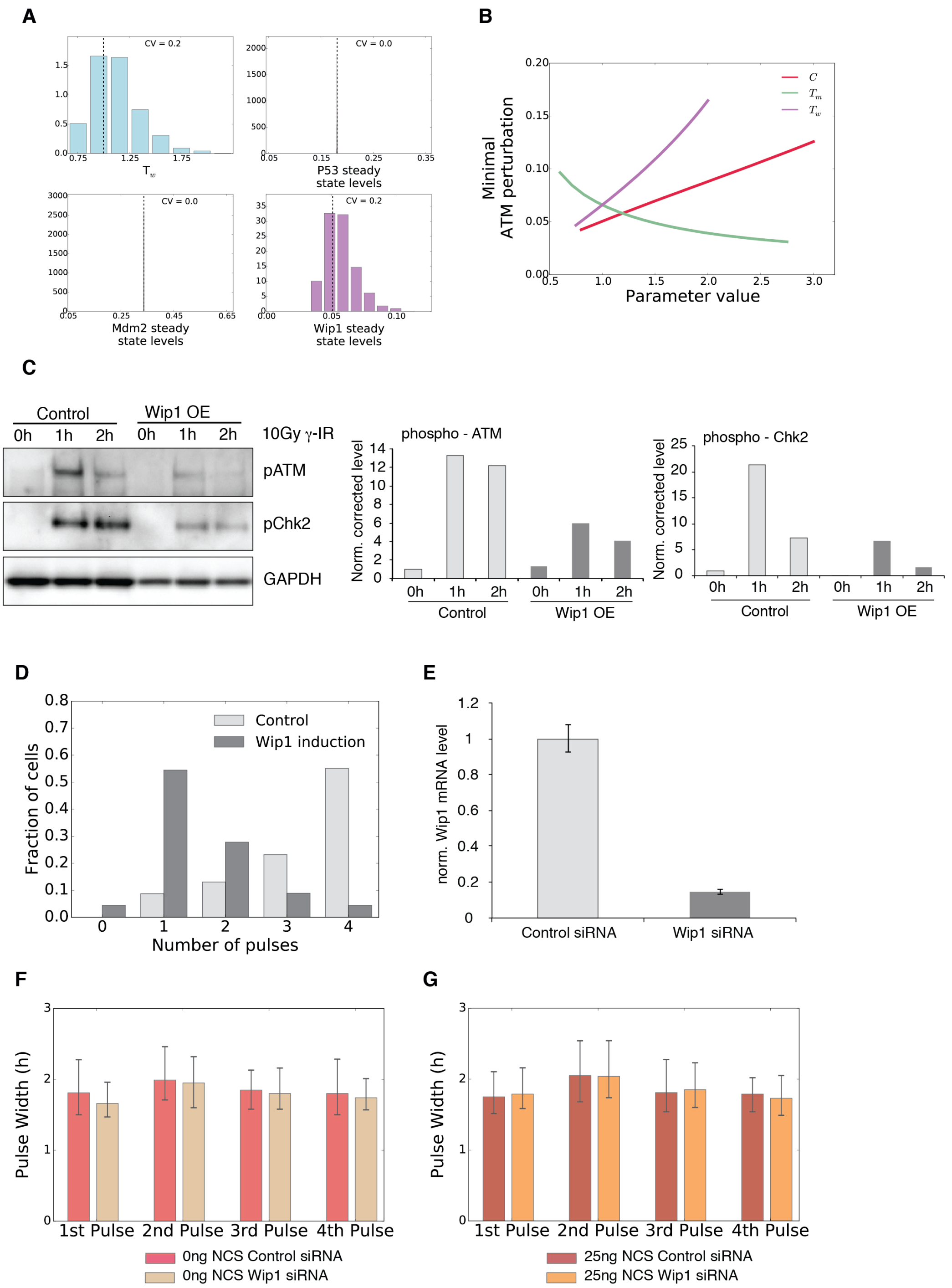

Figure S8 – Modeled Wip1 variability

A The assumed log-normal distribution of the Wip1 transcription rate (T_W) and the corresponding protein distributions at steady state. Wip1 mRNA levels are practically decoupled from the network under basal conditions, only influencing Wip1 Wip1 protein.

B Sensitivity scans for the production rate of p53 protein, Mdm2 mRNA and Wip1 mRNA. The parameters were varied individually and for each value the new steady state was calculated. The corresponding minimal ATM perturbation needed to cross the excitation threshold was determined by simulation.

C Western blot analysis of ATM and Chk2 phosphorylation upon DNA damage in A549 cells overexpressing Wip1 compared to control cells. Quantification was performed by densitometry; signal intensities were corrected for unequal loading using GAPDH and normalized to undamaged control cells (Control 0h).
D Wip1 induction for highly (10Gy) stimulated cells. The number of pulses is clearly lower for cells with increased Wip1 expression.
E Mean Wip1 mRNA levels upon transfection of scrambled control and Wip1-targeting siRNA were measured by quantitative RT-PCR (3d after transfection). Wip1 expression was downresgulated below 20% of control levels. Error bars indicate standard deviation.
F and **G** Median pulse width distribution for cells with no (**E**) or low 25ng NCS (**F**) stimulation upon transfection with scrambled control or Wip1-targeting siRNA, error bars indicate the 1st and 3rd quartile respectively. The siRNA treated cells show no altered pulse widths compared to cells expressing normal levels of Wip1.

Excitability in the p53 network mediates robust signaling with tunable activation thresholds in single cells

Supplementary Information

Gregor Moenke, Elena Cristiano, Ana Finzel,
Dhana Friedrich, Hanspeter Herzel,
Martin Falcke and Alexander Loewer

Contents

1 Toy model equations 3

2 Tables of parameters for the toy models 4

3 p53 model equations 4

4 Table of parameters for the p53 model 6

5 Detailed description of the Markovian DSB process 6

5.1 The time-homogeneous repair process 7

5.2 Modeling the DSB induction by NCS 8

6 Bifurcation analysis of the p53 model 10

1 Toy model equations

The negative-feedback (NF) system presented in Figure 2 of the main text is given by:

$$\begin{aligned} \frac{d}{dt}X &= C - d_X X - d_{YX} Y * \frac{X}{k_{YX} + X} \\ \frac{d}{dt}y &= T_y \frac{X}{1+X} - d_y y \\ \frac{d}{dt}Y &= T_Y y - d_Y Y \end{aligned} \tag{1}$$

This network was inspired by simple models used to describe the p53-Mdm2 core negative feedback loop, for example as in Ref. [1]. The variable y describes an mRNA species whose production is activated by a protein X acting as transcription factor. The negative feedback closes with the degradation of X by the maturated protein Y.

The negative-positive feedback (NPF) system is given by the equations:

$$\begin{aligned} \frac{d}{dt}X &= \frac{C + X^2}{1 + X^2} - d_{YX} * X * Y - d_X * X \\ \frac{d}{dt}y &= T_y \frac{X}{1+X} - d_y * y \\ \frac{d}{dt}Y &= T_Y y - d_Y Y \end{aligned} \tag{2}$$

Here species X exhibits a self-activating term with a Hill coeffecient of two acting as the positive feedback, e.g. describing processes like auto-phosphorylation. This term alone would introduce bistability into the system. The additional negative feedback is topologically the same as for the NF system presented above, albeit the degradation of X by Y follows mass action kinetics. This NPF system resembles a so-called *bistable frustrated unit* as discussed by the authors of Ref. [2]. The parameters for both models are given as tables in section 2.

2 Tables of parameters for the toy models

Table 1: Negative feedback system model parameters

name	value
C	4.5
d_X	0.01
d_{YX}	7
k_{YX}	0.04
T_y	0.35
d_y	0.4
T_Y	0.67
d_Y	0.25

Table 2: Positive-negative feedback system model parameters

name	value
C	0.015
d_{YX}	0.14
d_X	0.1
T_y	0.21
d_y	0.1
T_Y	0.3
d_Y	0.1

3 p53 model equations

The core deterministic p53 model was formulated as a system of coupled ordinary differential equations (ODEs). The associated regulatory network is shown in Figure 3A of the main text. We modeled only the amount of activated ATM (ATM^*), where the positive feedback described in the main text is modeled phenomenologically by a self-activation term with a Hill coeffecient of two. The phosphatase Wip1 not only dephosphorylates ATM^* directly, but also other species (i.e. γH2AX, MRN complex) which are involved in the positive feedback [3]. This is modeled by an inhibition of the ATM^* self-activation term by the Wip1 protein. The constitutive expression of p53 (Figure S2A) was described by the constant production rate C for the protein $P53$. The p53 protein itself is a target of ATM^* and $Mdm2$ is reported to have a lower binding affinity to phosphorylated $P53$. As with the present data phosphorylated p53 was not quantified, no additional species was introduced into the model. Therefore,

this mechanism was also indirectly captured phenomenologically by inhibiting the $Mdm2$ dependent $P53$ degradation via ATM^* . The relative strength of this second ATM^* interaction is given by the parameter R. Transcriptional activation of the two mRNA species $mdm2$ and $wip1$ by $P53$ were modeled by saturating kinetics. All other terms of the ODE system follow mass-action kinetics or first-order kinetics. The dependence of the effective input signal S on the number of DSBs is modeled by a saturating kinetic to control both the maximal stimulation and the sensitivity towards DSBs. For the stochastic forcing (Figure 3D,E and F, main text) the number of DSBs is time-dependent and given by the stochastic DSB process defined below in section 5. The naming of the parameters follows general conventions: parameters containing the letter "T" denote production rates concerning translation or transcription, parameters starting with a "d" denote degradation rates and parameters containing "k" are the respective Michaelis constants. The subscripts encode the affiliation to the modeled species, e.g. a "m" in the subscript is associated with the Mdm2 mRNA and a "M" with the respective protein species. Thus the parameter k_{Pm}, for example, describes the Michaelis constant of the Mdm2 mRNA production induced by the p53 protein. Rate parameters which do not follow this nomenclature are: A which gives the maximal activation rate of ATM, P which describes the dephosphorylation of ATM^* by $Wip1$, g which gives the maximal $Mdm2$ dependent degradation of $P53$ and finally C which is the maturation rate of new $P53$ entering the system. An overview of all model parameters and their values is given in the table 4 below.

$$\begin{aligned}
\frac{d}{dt}ATM^* &= A\frac{ATM^{*2}}{1+ATM^{*2}/k_A}\frac{1}{1+Wip1/k_{WA}} - d_A ATM^* - P\ ATM^*\ Wip1 + S(DSB) \\
\frac{d}{dt}P53 &= C - d_P\ P53 - \ g\ Mdm2\ \frac{P53}{k_{MP}+P53}\left(1+\frac{R}{1+ATM^*}\right) \\
\frac{d}{dt}mdm2 &= T_m\frac{P53}{k_{Pm}+P53} - d_m\ mdm2 \\
\frac{d}{dt}Mdm2 &= T_M\ mdm2 - d_M\ Mdm2 - d_{AM}\ ATM^*\ Mdm2 \\
\frac{d}{dt}wip1 &= T_w\frac{P53}{k_{Pw}+P53} - d_w\ wip1 \\
\frac{d}{dt}Wip1 &= T_W\ wip1 - d_W\ Wip1 \\
S(DSB) &= S_{max}\frac{DSB}{(\gamma+DSB)}
\end{aligned} \tag{3}$$

4 Table of parameters for the p53 model

Table 3: Overview of all model parameters

name	meaning	value
A	maximal self activation rate of ATM	30.5
P	rate of dephosphorylation of ATM by $Wip1$	22
C	production rate of $p53$	1.4
g	maximal degradation of $P53$ by $Mdm2$	2.5
d_{AM}	degradation rate of $Mdm2$ by ATM^*	20
T_m	maximal production rate of $mdm2$	1
T_M	maximal production rate of $Mdm2$	4
T_w	maximal production rate of $wip1$	1
T_W	maximal production rate of $Wip1$	1
d_A	basal dephosphorylation rate of ATM^*	0.16
d_P	basal degradation rate of $P53$	0.1
d_m	basal degradation rate of $mdm2$	1.
d_M	basal degradation rate of $Mdm2$	2.
d_w	basal degradation rate of $wip1$	1.3.
d_W	basal degradation rate of $Wip1$	2.3
k_A	Michaelis constant for the ATM^* self activation	0.5
k_{WA}	Michaelis constant for the inhibition of the ATM^* self activation by $Wip1$	0.14
k_{MP}	Michaelis constant for the degradation of $P53$ by $Mdm2$	0.15
k_{Pm}	Michaelis constant for the production of $mdm2$ by $P53$	1.
k_{Pw}	Michaelis constant for the production of $wip1$ by $P53$	1.
R	strength of the inhibition of the $Mdm2$ mediated degradation of $P53$ by ATM^*	2
S_{max}	maximal signal strength of the DSB process	0.2
γ	Michaelis contant for the signal strength of the DSB process	9

5 Detailed description of the Markovian DSB process

The dynamics of the DNA double strand breaks (DSBs) were considered as the primary input of the p53 model. Fortunately, as outlined in the main text, single cell trajectories of damage foci are available. While the direct relation between foci number and number of DSBs remains unclear, they can still serve as a quantitative marker [4]. Therefore, the number of foci was treated as the number of DSBs. Typically the DSB dynamics are described by an exponential model [5], which sufficiently describes the repair process. However, the half-lifes of the foci show large variability across a cell population [4, 5]. The sources of the observed stochasticity of the DSB dynamics lie both within the processes which cause the DNA damage as well as the cellular repair mechanisms. The former include e.g. the errors arising from mitotic division, the occurrence of radical metabolic byproducts and cosmic radiation, which are intrinsically noisy. The

latter appear to be irregular also due to various reasons, e.g. different severity of the DNA lesions or different copy numbers and spatial availability of the proteins involved in the repair process.

5.1 The time-homogeneous repair process

A simple approach to model the stochastic repair process is to define two rates b and r by the following assignments of probabilities, given that there are n DSBs present at time t:

$$\begin{aligned} b\ dt &= \textit{probability that a new DSB occurs in } [t, t+dt] \\ nr\ dt &= \textit{probability that a DSB is repaired in } [t, t+dt]. \end{aligned} \tag{4}$$

This is a Markovian birth-death process, named the *payroll process* by Gillespie [6]. The asymptotic analytical solutions for the mean and variance read:

$$\begin{aligned} \lim_{t\to\infty} \langle DSB(t) \rangle &= N_b = \frac{b}{r}, \qquad \textit{and} \\ \lim_{t\to\infty} Var\big(DSB(t)\big) &= \frac{b}{r}. \end{aligned} \tag{5}$$

These relations only provide the ratio between the two rates b and r. To effectively reverse calculate the rates from the foci data, dynamical properties of the process have to be taken into account. The aim here is not to claim or provide an exact parameter estimation, but to give a reasonable estimation given the simplistic stochastic model used and the data available. In the following, the background damage level N_b is used for the estimation, this already gives $b = N_b r$. The first moment time-evolution function for the payroll process given in ref. [6] reads:

$$\begin{aligned} \langle DSB \rangle_t &= e^{-rt}\left(N_0 + \int_0^t b e^{rt'} dt'\right) \\ &= \left(N_0 - \frac{b}{r}\right) e^{-rt} + \frac{b}{r}. \end{aligned} \tag{6}$$

Substituting with the asymptotic mean yields

$$\langle DSB \rangle_t = (N_0 - N_b) e^{-rt} + N_b. \tag{7}$$

The notion of N_b and $\langle N_b \rangle$ might be a little confusing. For the stochastic process, these two are identical, N_b being the asymptotic time average of one realization and $\langle N_b \rangle$ being the asymptotic ensemble average. For the time series foci data, these two hardly coincide. Reasons for that are the finite and rather short period of sampling, some additional cell-to-cell variability and probably some systematic error in the measurements, as the number of foci is only a proxy for the number of DSBs. Nevertheless, to advance with the estimation, the l.h.s. of equation 7 is treated as an ensemble average at time t. Solving for

the repair rate one obtains:

$$r = t^{-1} ln \left(\frac{N_0 - N_b}{\langle DSB \rangle_t - N_b} \right) \tag{8}$$

The time dependence of the rate is obviously artificial, as the rate should be time independent for a time homogeneous process. And indeed, when using this formula to reverse calculate the repair rate out of an ensemble of synthetic data, r is time independent for all $0 < t < t_c$. An example is shown in figure S4A and B. However, when using equation 8, one carefully has to observe the denominator. From a critical time on, the ensemble average at time t_c gets very close to the asymptotic average: $\langle DSB \rangle_{t_c} \approx N_b$. This quickly leads to numerical precision issues, as the denominator converges exponentially fast to zero and might even turn negative due to fluctuations. Practically this means, that the estimation of the repair rate r should be carried out in a time domain, where the trajectories on average are still decaying. To improve the estimate, an averaging within the respective time domain $[0, t_c]$ can be carried out and was done for the inference from the real data. It is noteworthy, that the relaxation dynamics are needed for the parameter estimation. An unstimulated ensemble of trajectories corresponding to stationary dynamics alone would not allow for the described method as this would correspond to $t_c = 0$.

Having demonstrated that the proposed method for rate parameter estimation works with synthetic data, a dataset containing cells irradiated with 5Gy of gamma radiation was analyzed accordingly. As the DSB induction for irradiated cells is very fast, only the repair dynamics are observable. Because every foci trajectory has generally a different N_0, the data is binned to form subensembles with comparable initial amount of DSBs. As can be seen in figure S4C, the initial amount of DSBs present in the cells has a broad distribution despite the population received a fixed damage dose. This makes the parameter estimation even more difficult, due to the low sample numbers in the subensembles. The results for two different subensembles for the 5Gy data set are shown in figure S4D. Given the broad initial distribution and that there are only 63 cells in total, there is no satisfactory pooling available. But nevertheless, the repair rate was estimated to be in the order of $r \approx 0.315h^{-1}$ per hour, which yields an average lifetime of a damage locus to be around 3 hours (Figure S4E). This is in good agreement with the results reported experimentally [4, 5]. Given the mean damage background level to be $\langle N_b \rangle \approx 2.3$, the corresponding basal break rate for the DSB process is $b \approx 0.8h^{-1}$.

5.2 Modeling the DSB induction by NCS

The dataset used for the main results in the main text consists of cells which were stimulated with the radiomimetic drug neocarcinostatin (NCS). As opposed to irradiated cells, the DSB induction is directly observable and lasts around an hour in these experiments (Figure 3 B and C). To address this phase of rapid DSB induction within our Markovian modeling framework, we augmented the stochastic process with a time dependent break rate:

$$b(t) = \begin{cases} b_s, \ if \ t \leq T_s \\ b_b, \ if \ t > T_s \end{cases} . \tag{9}$$

Here b_b is the basal break rate as estimated in section 5.1 above, and $b_s > b_b$ is the NCS dose dependent stimulation break rate. The switching between both rates occurs at T_s.

To devise a simulation algorithm we first recall the *next-jump density function* from the classical Gillespie algorithm (SSA) [6]:

$$\rho(\tau|n,t) = \sum_k a_k(n, t+\tau) exp\left(-\sum_k \int_0^\tau a_k(n, t+\tau')d\tau'\right), \tag{10}$$

where the $a_k's$ are the propensities. Using integration by parts and employing inversion sampling gives the expression:

$$\sum_k \int_0^\tau a_k(n, t+\tau')d\tau' = ln(1/r), \tag{11}$$

here r is a random number drawn from the uniform distribution $U(0,1)$. Solving this equation for τ allows for the SSA algorithm. For the time-homogeneous case $a_k(n,t) \equiv a_k(n)$ this is straightforward:

$$\tau_{hom} = ln(1/r)/\sum_k a_k(n) = ln(1/r)/(b_b + rn), \tag{12}$$

where for the last expression we substituted the propensities for our homogeneous DSB process. For arbitrary time-inhomogeneous processes equation 11 often has to be solved numerically, which greatly increases the cost of the algorithm. However, as our time-inhomogeneous rate $b(t)$ is a simple step-function, equation 11 can still be solved analytically. Integration yields a family of piecewise linear functions and depending on the systems time t the next jump occurs at:

$$\tau_{inhom} = \begin{cases} z/(b_s + rn), \ if \ t \leq T_s \ and \ z \leq (b_s + rn)\Delta_s \\ \left[z - (b_s - b_b)\Delta_s\right]/(b_b + rn), \ if \ t \leq T_s \ and \ z > (b_s + rn)\Delta_s \\ z/(b_b + rn), \ if \ t > T_s, \end{cases} \tag{13}$$

here $\Delta_s = T_s - t$ is the distance in systems time to the break rate switch and $z = ln(1/r)$. The additional conditions on z for the first two cases ($t \leq T_s$) ensure, that the correct section of the piece wise linear function is inverted.

In summary, augmenting the cost effective SSA algorithm with a test for two conditions per step makes it readily applicable to time dependent rates formulated as step functions. An example realization of the DSB process upon NCS stimulation is shown in Figure S4F. The good agreement of our DSB process with an ensemble of NCS stimulated cells is shown in Figure 3C of the main text.

6 Bifurcation analysis of the p53 model

The one parameter (or codimension one) bifurcation analysis of the deterministic p53 model is shown in Figure S3A. The control parameter is the signal strengths S which appears on the r.h.s. of the equation for ATM^* (see section 3), describing DSB induced ATM activation. For a low input signal, equivalent to no or very low damage levels, the system is in the excitable regime. This is characterized by the presence of one stable and two unstable fixed points or steady states. The lowest fixed point is a stable spiral, giving rise to small subthreshold oscillations for small perturbations. The middle fixed point is a saddle, whose stable manifold separates the phase space into regions of different qualitative behavior. This *separatrix* acts as an direction dependent threshold for the system. The bifurcation leading to the oscillations in the p53 model is the saddle-node homoclinic bifurcation [7]. This is a global bifurcation involving a local saddle-node bifurcation. What happens at the bifurcation point is, that the heteroclinic connection of the saddle to the stable fixed point becomes a homoclinic orbit of the merged saddle-node. This saddle-node then disappears via the local saddle-node bifurcation (denoted as limit point in figure S3A) and a limit cycle appears near the former homoclinic orbit (called a *saddle node on invariant circle* (SNIC) bifurcation). From within the excitable regime, which is considered as operating point of the model for no DSBs present, the oscillatory regime can only be reached by increasing the signal S. The limit cycles of this positive feedback oscillator are born with huge amplitudes, and show only very little dependence on the signal strength up to $S \approx 0.5$. In effect, for no or low signal the system resides in an excitable state capable of showing isolated pulses. In addition, for a stronger signal after the saddle-node homoclinic bifurcation the system undergoes stable sustained oscillations with pulse shapes very similarto excitory pulses (Figure 3D-F main text). These are exactly the characteristics found in the p53 single cell data for low vs. high damage input, which were discussed in the main text.

To further extend the bifurcation analysis, a search for codimension-2 bifurcations was performed as shown in figure S3B. Here a Cusp bifurcation point (CP) was found. This marks the appearance of the phasespace structure required for the excitability class I regime: the co-existence of a saddle as organizing center, one stable and one unstable fixed point. There is additionally a Bogdanov-Takens (BT) point very close by, spawning a Hopf bifurcation curve which effectively destabilizes the upper fixed point and makes the system excitable. The specific bifurcation parameters used are T_W, the Wip1 protein production rate, and again S. However, there are actually only 2 symmetric types of these codimension-2 diagrams, the other type and further examples are presented in Ref. [8]. The actual region of excitability in parameter space extends beyond the three fixed point regime (see figure S3B). Although the two upper fixed points vanished via a saddle-node bifurcation, the phase space still has *memory* about their presence. What is meant by that is, that the global flow (the excitation loop) is not seriously affected by this local bifurcation. The exact definition of the threshold as separatrix is, however, lost in this regime. The oscillatory regime here is reached via a subcritical Hopf bifurcation fol-

lowed by a saddle-node bifurcation of limit cycles. The stable limit cycle again suddenly appears with huge amplitudes.

Notably, besides the excitable and oscillatory regimes there is a bistable regime for very low maximal transcriptional activation of Wip1 and a not too strong signal. This is due to the now weak negative feedback which is facilitated by the Wip1-ATM interaction. Therefore the positive feedback predominates and induces bistability. Interestingly, for medium to high signal strengths the bistability is lost via a saddle-node bifurcation and the system becomes monostable, with the highest steady state now being the only attractor of the system. This resembles the behavior found for Wip1 knockout cells [9].

References

[1] N. Geva-Zatorsky *et al.*, Molecular systems biology **2** (2006).

[2] S. Krishna, S. Semsey, and M. H. Jensen, Physical biology **6**, 036009 (2009).

[3] H. Yamaguchi, S. R. Durell, D. K. Chatterjee, C. W. Anderson, and E. Appella, Biochemistry **46**, 12594 (2007).

[4] A. Loewer, K. Karanam, C. Mock, and G. Lahav, BMC biology **11**, 114 (2013).

[5] K. Karanam, R. Kafri, A. Loewer, and G. Lahav, Molecular cell **47**, 320 (2012).

[6] D. T. Gillespie, *Markov processes: An introduction for physical scientists* (Elsevier, 1991).

[7] Y. A. Kuznetsov, *Elements of applied bifurcation theory* (Springer New York, 1995).

[8] G. Moenke, *Stochastic oscillations in living cells*, PhD thesis, Humboldt-Universität zu Berlin, Lebenswissenschaftliche Fakultät, 2015.

[9] E. Batchelor, C. S. Mock, I. Bhan, A. Loewer, and G. Lahav, Molecular cell **30**, 277 (2008).
